# Cassava *shrunken-2* homolog *MeAPL3* determines storage root starch and dry matter content and modulates storage root postharvest physiological deterioration

**DOI:** 10.1007/s11103-020-00995-z

**Published:** 2020-10-06

**Authors:** Getu Beyene, Raj Deepika Chauhan, Jackson Gehan, Dimuth Siritunga, Nigel Taylor

**Affiliations:** 1 Donald Danforth Plant Science Center, St. Louis, MO USA; 2 Department of Biology, University of Puerto Rico, Mayaguez, Puerto Rico

**Keywords:** Cassava, ADP-glucose pyrophophorylase, *MeAPL3*, Starch, Dry matter, Postharvest physiological deterioration

## Abstract

**Key message:**

Among the five cassava isoforms (*MeAPL1*–*MeAPL5*), MeAPL3 is responsible for determining storage root starch content. Degree of storage root postharvest physiological deterioration (PPD) is directly correlated with starch content.

**Abstract:**

AGPase is heterotetramer composed of two small and two large subunits each coded by small gene families in higher plants. Studies in cassava (*Manihot* *esculenta*) identified and characterized five isoforms of *Manihot* *esculenta* ADP-glucose pyrophosphorylase large subunit (*MeAPL1*–*MeAPL5*) and employed virus induced gene silencing (VIGS) to show that *MeAPL3* is the key isoform responsible for starch and dry matter accumulation in cassava storage roots. Silencing of *MeAPL3* in cassava through stable transgenic lines resulted in plants displaying significant reduction in storage root starch and dry matter content (DMC) and induced a distinct phenotype associated with increased petiole/stem angle, resulting in a droopy leaf phenotype. Plants with reduced starch and DMC also displayed significantly reduced or no postharvest physiological deterioration (PPD) compared to controls and lines with high DMC and starch content. This provides strong evidence for direct relationships between starch/dry matter content and its role in PPD and canopy architecture traits in cassava.

**Electronic supplementary material:**

The online version of this article (10.1007/s11103-020-00995-z) contains supplementary material, which is available to authorized users.

## Introduction

Cassava (*Manihot esculenta* Crantz) is widely grown for its starchy storage roots across the tropics and sub-tropics, with an estimated annual production of over 292 million metric tonnes (MT) in 2017 (FAOSTAT, accessed 12/17/2019). Africa accounts for 61% of this production, with Nigeria being the world’s largest producer at 59.4 million MT. Cassava is grown by 100 s millions of smallholder farmers as a subsistence crop. However, dependence on the crop in sub-Saharan Africa is threatened by biotic stresses, especially cassava mosaic disease (CMD), cassava brown streak disease (CBSD), cassava bacterial blight (CBB) and cassava green mites (CGM) (Bull et al. 2011; Bart and Taylor 2017). Cassava production and utilization is also limited by inherent susceptibility of storage roots to rapid postharvest deterioration after removal from the soil (Beeching et al. 1998). This commences 24–48 h after harvest and is known as postharvest physiological deterioration (PPD). Globally, postharvest losses of cassava storage roots are estimated at about 19% (Zainuddin et al. 2018), and in Africa may reach 29% (Djabou et al. 2017). PPD has been classified as primary and secondary in nature. The former is considered enzymatic, and happens early in the process, while the latter involves mainly microbial deterioration of affected storage root tissues (Booth 1976). Studies have shown association of PPD with increased respiration and water loss (Marriott et al. 1978), elevated activities of enzymes involved in wound response, production of phenols and polyphenols (Rickard 1985) and changes in sugar and starch content (Booth 1976). Oxidative burst, believed to be an early event that leads to PPD, has been causally linked to the process of cyanogenesis (Zidenga et al. 2012). Studies of transcripts (Reilly et al. 2007), the proteome (Vanderschuren et al. 2014) and metabolites (Uarrota and Maraschin 2015) have documented potential genes and pathways involved in the progression of PPD. However, details of its molecular basis and the underlying sequential biochemical steps remain elusive, making development of affordable and sustainable mitigation measures an ongoing challenge.

Cassava is mainly grown for its starch-rich storage roots, although young leaves are consumed as a nutritious vegetable in some countries (Howeler et al. 2013). Preceded only by cellulose, starch is the most abundant polymer on earth (Geigenberger 2011) and used as food, feed, biofuel and in many industrial applications (Smith 2008; Geigenberger 2011; Streb and Zeeman 2012). Starch is the predominant metabolite in cassava storage roots, accounting for 65–91% on dry weight (DW) basis (Sánchez et al. 2009). Starch is synthesized and stored in the chloroplasts of photosynthetic organs in a transitory manner, and in the longer term in amyloplasts of heterotrophic tissues such as cassava storage roots, potato tubers and cereal endosperm (Streb and Zeeman 2012). The sequential steps and genes involved in starch metabolism have been elucidated in plants (Geigenberger 2011; Streb and Zeeman 2012). Computational approaches based on known plant starch metabolism genes have been used to document cassava genes potentially involved in starch metabolism (Saithong et al. 2013; Tappiban et al. 2019). This knowledge has been exploited to tailor the type and quality of starch produced in cassava storage roots (Raemakers et al. 2005; Zeeman et al. 2010; Bull et al. 2018; Wang et al. 2018). The first committed step in starch biosynthesis is catalyzed ADP-glucose pyrophosphoryalase (AGPase). AGPase is a heterotetramer, composed of two large and two small subunits in higher plants (Copeland and Preiss 1981). These enzymes are coded by a small gene family of two to three genes for the small subunit, and three to five genes for the large subunit in most species studied to date (Ballicora et al. 1998, 2005; Crevillén et al. 2003). The small subunit plays a catalytic role, while the large subunit plays both catalytic and regulatory roles. The catalytic role of the large subunit was demonstrated in rice large subunit 2 (Tuncel et al. 2014), and in *A. thaliana*, APL1 and APL2. This catalytic function has been lost in APL3 and APL4 (Ventriglia et al. 2008). The different isoforms of APL genes differ in their kinetic and regulatory properties conferred to the hetetrotetrameric enzyme. It has also been hypothesized that different hetrotetramers are formed in various plant tissues depending on tissue specific expression patterns of the different isoforms, thereby controlling the rate of starch synthesis in these organs (Crevillén et al. 2003).

The importance of AGPase in starch biosynthesis has been described through characterization of mutants. For example, the maize *brittle-2* (*bt2*) and *shrunken-2* (*sh2*) are mutants for the small and large subunits, respectively (Bhave et al. 1990; Bae et al. 1990). Rice mutants *osagps2-1* and *osagpl2-1*lack isoforms of the small (S2b) and large (L2) subunits (Lee et al.^2007^). Activated glucose (ADP-glucose) is the substrate used in starch biosynthesis, with synthesis of ADP-glucose from glucose-1-phosphate and ATP by AGPase considered to be the rate limiting step (Preiss 1984; Stark et al. 1992). The catalytic activities of AGPase are under allosteric regulation, whereby the net catalytic activity is increased by activator 3-phosphoglycerate (3-PGA) and inhibited by inorganic phosphate (Pi) (Ballicora et al.^2004^). Other mechanisms including redox, phosphorylation and transcriptional regulation of the tetramer have also been described (Geigenberger 2011).

In cassava, homologs of *bt2* and *sh2* have been cloned and their expression pattern determined by northern blot analysis in different tissues (Munyikwa et al. 2001). Presence of multiple isoforms of APL genes in cassava is expected (Saithong et al. 2013; Dong et al. 2019; Tappiban et al. 2019), and would be consistent with monocot and dicot plant species (Ballicora et al. 2004). In the present study, we identified five isoforms of cassava APL genes (*MeAPL1-MeAPL5*) from the cassava genome and characterized their functional significance in starch biosynthesis in storage roots using virus induced gene silencing (VIGS). Data is presented to show that among the five cassava APL isoforms, *MeAPL3* is critical for starch accumulation in cassava storage roots. Study of stable transgenic *MeAPL3* co-suppression lines displaying varying levels of storage root starch/dry matter content provide evidence for a strong and direct relationship between starch, dry matter content and cassava PPD. Reduction in starch and dry matter content was also associated with changes in plant architecture, notably petiole leaf angle.

## Materials and methods

### Identification of *MeAPL* genes, production of plasmid vectors and transgenic plants

Isoforms of cassava ADP-glucose pyrophophorylase large subunit (APL) genes *MeAPL1* (Manes.01G236700), *MeAPL2* (Manes.03G182100), *MeAPL3* (Manes.11G085500), *MeAPL4* (Manes.15G025400) (Dong et al. 2019; Tappiban et al. 2019) and *MeAPL5* found in the cassava genome as one loci, but represented by the two accessions, Manes.S107700 and Manes.18G019600 were identified and retrieved from the cassava v6.1 genome sequence database at phytozome (www.phytozome.jgi.doe.gov/pz/portal.html, Bredeson et al. 2016).

Virus induced gene silencing (VIGS) vectors were generated by modification of the DNA-A component of *East African cassava mosaic virus* (EACMV-K201) (Beyene et al. 2017b). VIGS constructs targeting the five *MeAPL* genes were generated using 451–452 bp products amplified from cDNA (see section ‘RNA extraction, RT-PCR and Real-time quantitative PCR’). Forward and reverse primer pairs (Supplementary Table 1) used to amplify the respective *MeAPL* genes carried the *Nhe* I and *Sbf* I restriction sites. Amplified PCR products were cloned into pCR-Blunt II-TOPO vector (Invitrogen) and verified by sequencing. DNA fragments were digested with *Nhe* I and *Sbf* I from the TOPO cloning vector and sub-cloned into *Nhe* I and *Sbf* I digested EACMV-K201 based VIGS vector as described by Beyene et al. (2017b). Cloned VIGS vectors were named MeAPL1-VIGS, MeAPL2-VIGS, MeAPL3-VIGS, MeAPL4-VIGS and MeAPL5-VIGS, respectively, after the corresponding genes targeted. In addition, a VIGS-vector (Patatin-VIGS) targeting the 421 bp 3′-region of the patatin promoter driving expression of both *crtB* and *DXS* transgenes in pEC20 lines (Beyene et al. 2018) was amplified by introducing restriction sites *Nhe* I and *Sbf* I as described above, and sub-cloned into the infectious EACMV-K201 based VIGS vector.

A modified pCAMBIA2300 binary vector was used for production of stable transgenic cassava plants. Modifications included replacement of the *npt*II plant selectable marker gene with hygromycin phosphotransferase (*hpt*II) driven by the *Agrobacterium tumefaciens* nopaline synthase promoter (NOS), with the resulting vector named p8384. The p8384 binary vector was used directly (empty-vector control) or after addition of an expression cassette of *MeAPL3* under control of the *Cauliflower mosaic virus* promoter (CaMV 35S) (Kay et al. 1987). The *MeAPL3* coding sequence was amplified from cDNA prepared from leaves of cassava variety TME 7S (Beyene et al. 2017b) using primers shown in Supplementary Table 1, with *Eco* RI and *Bam* HI sites introduced for subsequent sub-cloning. The amplified *MeAPL3* coding sequence was cloned into pCR-Blunt II-TOPO vector (Invitrogen) and verified by sequencing. The expression cassette harboring CaMV35S::MeAPL3::35S-terminator was assembled in an intermediate vector and moved to p8384. The resulting binary vectors named p8388 (CaMV 35S::MeAPL3::35S-terminator/p8384) and the empty vector control p8384 (NOS::HPTII::NOS-terminator) were electroporated into *A. tumefaciens* strain LBA4404 and used to transform friable embryogenic callus of the CMD-susceptible cassava cultivar TME 7S. Stable transgenic cassava plants were generated following Chauhan et al. (2015). Recovered transgenic plants were characterized for presence of the transgene by PCR using primers described in Supplementary Table 1, and positive lines propagated as in vitro plantlets and established in the greenhouse (Taylor et al. 2012).

### Inoculation with infectious VIGS clones

Four- to six-week-old greenhouse grown plants of the CMD-susceptible cassava cultivar TME 7S were inoculated with VIGS clones (Beyene et al. 2017b). Six to ten plants were inoculated with each of the VIGS vectors MeAPL1-VIGS (p8376), MeAPL2-VIGS (p8378), MeAPL3-VIGS (p8284), MeAPL4-VIGS (p8379) and MeAPL5 (p8532) and the Patatin-VIGS (p8310). In all cases, the DNA-A VIGS component was combined with the DNA-B component of EACMV-K201 and bombarded into the top three leaves of wild-type TME 7S and transgenic pEC20-08 and pEC20-11 (Beyene et al. 2018) using a Helios® Gene Gun (BioRad, Hercules, California) (Beyene et al. 2016). Approximately 70 ng each of the VIGS (DNA-A) and DNA-B components was used to inoculate each plant. A control vector in which the DNA-A carried a 453 pb targeting the green fluorescent protein (GFP-VIGS) (Beyene et al. 2017b) was included as a control.

### RNA extraction, RT-PCR and real-time quantitative PCR

Level of VIGS-mediated suppression of each targeted *MeAPL* gene and GFP control was quantified by RT-qPCR. Total RNA was extracted from leaves and storage root samples collected from plants after 16–17 weeks growth in the greenhouse. Fully expanded leaves were collected from the third through fourth positions below the shoot-tip and frozen immediately in liquid nitrogen. Storage root samples were obtained by cleaning harvested roots in running tap water, slicing transversely and peeling. Samples were placed in 50 mL Falcon tubes and immediately flash frozen in liquid nitrogen. Samples were then freeze dried and used for total RNA extraction (Beyene et al. 2018). Total RNA was extracted from ~ 50 mg of fresh frozen leaf or freeze-dried storage root powder using the cetyltrimethylammonium bromide (CTAB) method (Doyl and Doyle 1990). Genomic DNA was removed using the TURBO DNA-free Kit (Ambion) and synthesized cDNA used as template for RT-PCR and RT-qPCR. RT-qPCR analysis was performed following Ogwok et al. (2015). The cassava serine/threonine-protein phosphatase 2A (PP2A) housekeeping gene was used for normalization of expression values (Moreno et al. 2011). RT-qPCR primers used for quantification of targeted *MeAPL* genes are listed in Supplementary Table 1.

### Phenotypic assessment of greenhouse-grown plants

Leaf angle was measured at the junction of petiole and stem using a protractor 8–10 weeks after transfer to the greenhouse. Leaf/petiole angle was assessed on three leaves at positions 8, 9 and 10 nodes counted downwards from the top-most leaf. Three to four plants per transgenic line were assesssed. At harvest, 16–17 weeks after planting, data on storage root number and storage root fresh weight was collected.

### Dry matter, starch, sucrose and glucose determination

Storage root dry matter content was determined as described by Beyene et al. (2018). Free sugars (glucose, fructose, sucrose, lactose, maltose and raffinose) (Kakehi and Honda 1989) and starch (AACC 2000) determinations were performed at the Agricultural Experiment Station Chemical Laboratories (AESCL), University of Missouri-Columbia, USA, using approximately 200 mg of freeze-dried cassava storage root flour.

### Iodine staining of storage root

Iodine staining of cassava storage roots was performed using Lugol’s (iodine–potassium iodide, IKI) staining solution. Harvested storage roots were cut transversely into 1–2 cm thick sections. Sections were incubated in Lugol’s solution in a Petri dish for one minute and then rinsed with distilled water. Sections were tapped dry with a paper towel. Images were captured using an iPhone6 Plus.

### PPD determination

Greenhouse grown cassava plants were removed from pots 16 to 17 weeks after planting and storage roots were harvested by cutting with pruning shears at the stem/root junction. Storage roots were washed clean of soil debris under running tap water and blotted dry, making sure not to inflict wounds or bruises. Roots were placed in paper bags and stored at 25 °C day/night temperature and RH of 60% in the dark. Assessment for PPD was performed by slicing storage roots transversely at proximal, middle and distal ends to produce approximately 2 cm thick segments along the root length. PPD was assessed visually by assigning a score of 0–100% as described by Salcedo and Siritunga (2011).

## Results

### Genes coding for ADP-glucose pyrophosphorylase large subunit in cassava and their expression pattern

Multiple genes (isoforms) coding for ADP-glucose pyrophosphorylase large subunit (APL) exist in different plant species. Using known *Arabidopsis thaliana* APL amino acid sequences (AtAPL1, AtAPL2, AtAPL3 and AtAPL4) as bait (Crevillén et al. 2003, 2005), we identified five APL genes in v6.1 of the cassava genome sequence available at Phytozome (https://phytozome.jgi.doe.gov/pz/portal.html; Bredeson et al. 2016). These are named *MeAPL1- MeAPL5* for *M* *anihot* *e* *sculenta* ADP-glucose pyrophosphorylase large subunit 1–5. *MeAPL1* (Manes.01G236700), *MeAPL2* (Manes.03G182100), *MeAPL3* (Manes.11G085500), and *MeAPL4* (Manes.15G025400) were present as full-length, and named as such in a recent report (Dong et al. 2019). *MeAPL5* was represented by two partial sequences with accession numbers Manes.S107700.1 g and Manes.18G019600.1 g, that share partial overlaps of about 500 bp. These represent apparent 5′- and 3′- regions respectively, of the same predicted gene sequence (data not shown). The contig from the coding regions of the two partial sequences had an ORF of 529 aa that shares high sequence identity (80–86%) with other plant APL genes of closely related species rubber (*Hevea brasiliensis*), castor bean (*Ricinus communis*) and black cottonwood (*Populus trichocarpa*).

**Fig. 1:**
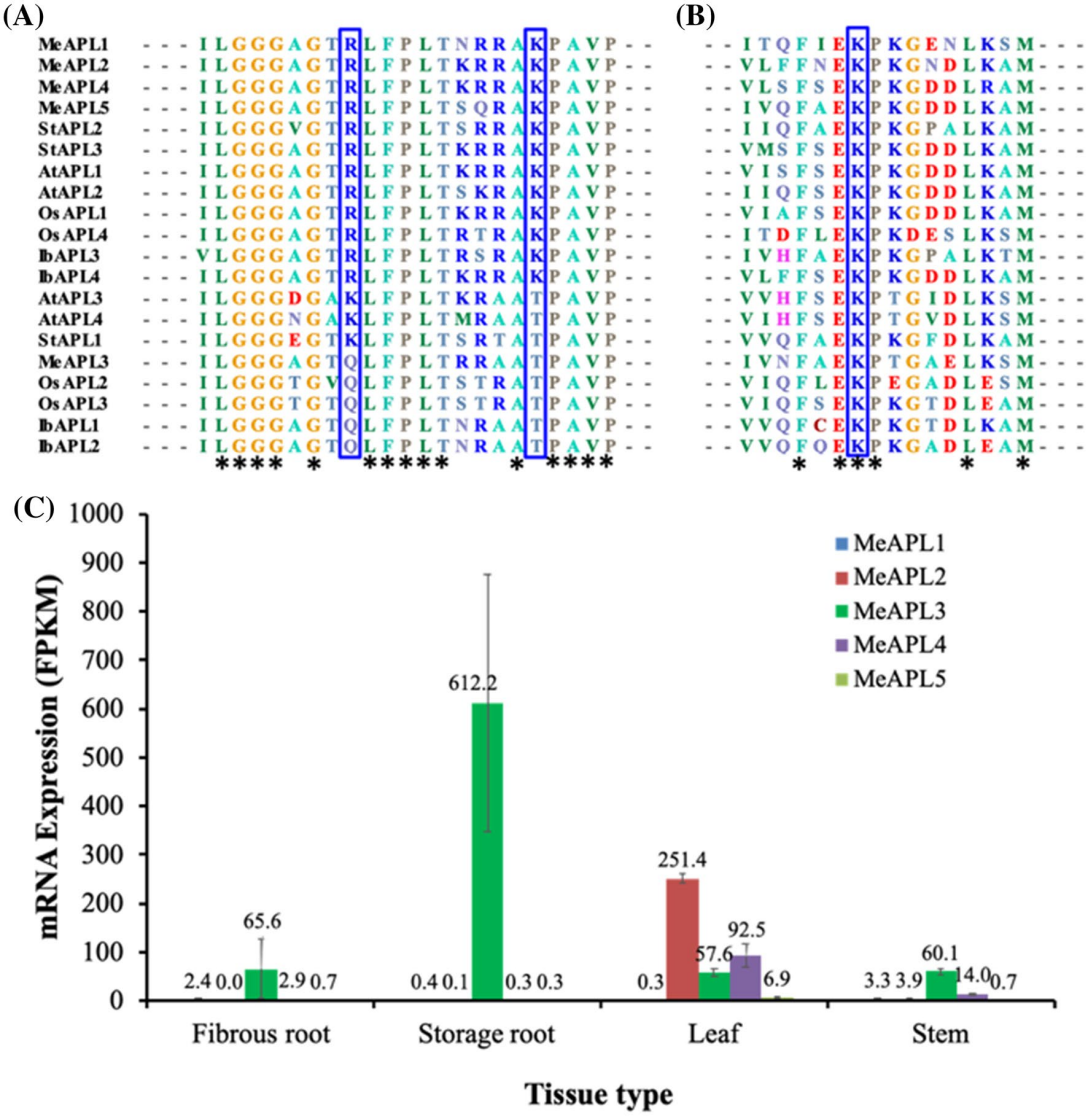
Amino acid sequences of cassava and selected plant ADP-glucose pyrophophorylase large subunit (APL) conserved regions and expression patterns in major organs. **a** Amino acid sequences of catalytic site, **b** amino acid sequences of Glucose-1-Phospahte (Glu-1-P) binding region. The conserved amino acid residues [R/K/ or Q] at position 102 and [K/T] at position 112 (amino acid position based on AtAPL1 numbering) in catalytic sites in **a** and conserved [K] residue at position 271 in Glu-1-P binding site in **b** are shown in rectangular boxes. *indicates conserved amino acid residues across the 20 APL genes. Accession numbers cassava *MeAPL1* (Manes.01G236700), *MeAPL2* (Manes.03G182100), *MeAPL3* (Manes.11G085500), *MeAPL4* (Manes.15G025400), *MeAPL5* (GenBank MN734216); Arabidopsis AtAPL1 (AT5G19220), AtAPL2 (AT1G27680), AtAPL3 (AT4G39210), AtAPL4 (AT2G21590); Sweet potato IbAPL1 (AFL55396); IbAPL2 (AFL55397), IbAPL3 (AFL55398), IbAPL4 (AFL55399); Potato StAPL1 (XP_006365120), StAPL2 (XP_015163910), StAPL3 (NP_001275395) and rice OsAPL1 (Q6AVT2), OsAPL2 (Q7G065), OsAPL3 (Q688T8) and OsAPL4 (Q0D7I3) (**c**) expression pattern of the five cassava APL genes in selected organs extracted from cassava expression atlas (Wilson et al. 2017) showing abudance of *MeAPL3* in storage roots. Bars show SD (*n* = 2–3)

To confirm that *MeAPL5* is coded by a single loci, cDNA covering the coding sequence and corresponding genomic region was amplified and sequenced from cassava variety TME 7S. This sequence has been deposited under GenBank MN734216 for cDNA and MN734217 for genomic DNA to represent *MeAPL5*. Coding sequences of the five *MeAPL* genes share 62–89% identity at the nucleotide level and 58–90% deduced amino acid sequence identity (data not shown). All *MeAPL* genes identified have conserved domains shared by *APL* genes of other plant species, as determined by searching the conserved domain database (CDD) available at NCBI (https://www.ncbi.nlm.nih.gov/Structure/cdd/wrpsb.cgi) (Marchler-Bauer et al. 2014) and reported by Dong et al. (2019). Alignment of the deduced amino acid sequences of the five cassava APL genes with APL from other plant species revealed conserved amino acid residues Arginine (R), Lysine (K) or Glutamine (Q) at position 102, and K or Threonine (T) at position 112 (numbering based on Arabidopsis APL1 amino acid) that are responsible for catalysis (Fig. 1a), and conserved amino acid K at position 271 (Fig. 1b) within Glucose-1-phophate (Glc-1-P) binding sites (Ballicora et al. 2005; Ventriglia et al. 2008).

Preferential expression of the five *MeAPL* genes in fibrous roots, storage roots, stems and leaves was assessed using the cassava ATLAS (https://shiny.danforthcenter.org/cassava_atlas) (Wilson et al. 2017). Expression of *MeAPL1* was minimal (0.2–3.3 FPKM) in fibrous roots, storage roots, stem and leaf tissues, while *MeAPL2*, *MeAPL4* and *MeAPL5* are preferentially expressed in leaves, with minimal or no expression in storage roots (Fig. 1c). *MeAPL3* was clearly the most expressed isoform in cassava storage roots, fibrous roots and stems, and found abundantly expressed at 10-times higher levels (612 FPKM) in storage root tissues (Fig. 1c).

### EACMV-VIGS is efficacious for suppressing gene expression in cassava storage roots

**Fig. 2:**
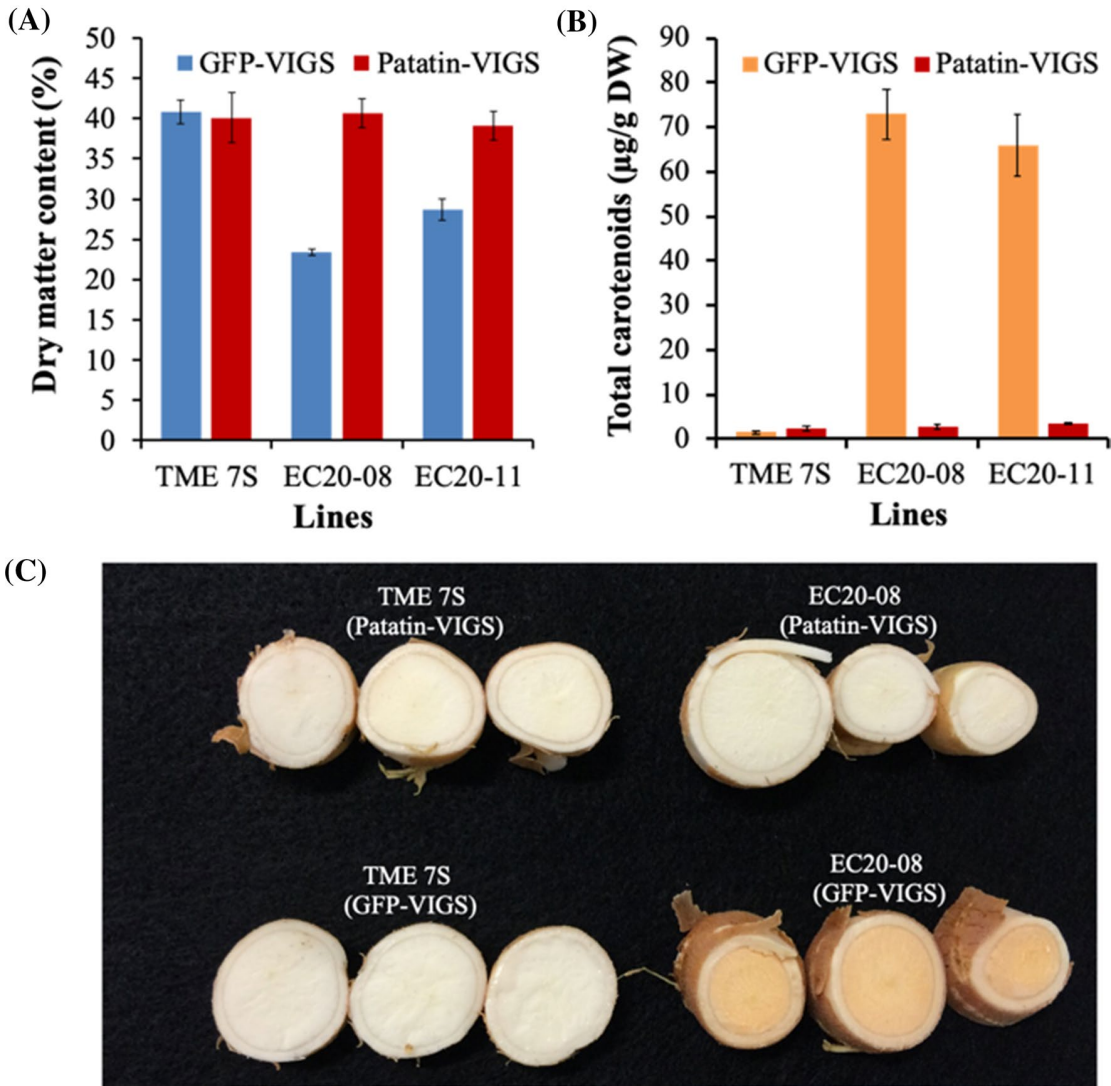
Functionality of *East African cassava mosaic virus* (EACMV-K201) based virus induced gene silencing (VIGS) in cassava storage roots. **a **Dry matter, **b** total carotenoid content and **c** transverse slices of transgenic storage roots harvested from EC20 plants and wild-type TME 7S after challenge with GFP-VIGS and Patatin-VIGS construct. EC20-08 and EC20-11 transgenic events co-express *crtB* and *DXS* transgenes, each driven by the patatin promoter (Beyene et al. 2018). Silencing of *crtB* and *DXS* expression resulting from targeting the patatine promoter with Patatin-VIGS abolishes carotenoid accumulation in storage roots (**b**, **c**) and restores DMC to wild-type levels (**a**), GFP-VIGS has no target in the cassava genome and does not affect carotenoid accumulation or DMC. Bars show SD (*n* = 4–6)

We previously showed functionality of VIGS in cassava based on use of a virulent infectious clone of the *East African cassava mosaic virus* (EACMV-K201) (Beyene et al. 2017b). VIGS-mediated gene silencing in shoot tissues was achieved for cassava phytoene synthase (*MePSY*) (Beyene et al. 2017b) and cassava *SPINDLY* (*MeSPY*), generating a visible phenotype on leaves and shoot-tips respectively (Beyene et al. 2017b). In order to demonstrate the ability of EACMV-based VIGS to silence genes in cassava storage roots, we utilized cassava plants accumulating provitamin A resulting from co-expression of *DXS* and *crtB* transgenes (Beyene et al. 2018). In these plants expression of both transgenes is driven by the predominantly storage organ-specific patatin promoter, resulting in orange coloration of storage root parenchyma, elevated ß-carotene and concomitant reduction in dry matter content (Beyene et al. 2018). Silencing *DXS* and *crtB* by targeting the patatin promoter using Patatin-VIGS in transgenic plant lines EC20-08 and EC20-11 abolished accumulation of carotenoids in storage roots (Fig. 2). Storage roots of the two transgenic lines inoculated with the control vector, GFP-VIGS, which has no target in the cassava genome, remained orange colored, while storage roots of the two transgenic lines challenged with Patatin-VIGS were white in color, in a manner similar to the non-transgenic wild-type control (Fig. 2c). Patain-VIGS-mediated suppression of transgene expression was further supported by measurement of total carotenoids and dry matter content (DMC) (Fig. 2a, b). Silencing of *crtB* and *DXS* by targeting the patatin promoter reduced total carotenoids from 73 and 66 ppm to 2.0 and 2.4 ppm, respectively, in plants of EC20-08 and EC20-11, a level comparable to that seen in non-modified wild-type TME 7S (Fig. 2b). As expected, VIGS-mediated silencing of *crtB* and *DXS* also resulted in elevation of storage root DMC to wild-type levels, increasing from 23.0% to 40.7% in plants of EC20-08, and from 28.6 to 39.0% in EC20-11. The DMC of wild-type TME 7S remained unchanged at 40% in plants challenged with GFP-VIGS and Patatin-VIGS (Fig. 2a). With effectiveness of the EACMV-K201 based VIGS shown for functional studies of gene expression in cassava storage roots, this technique was employed to target and downregulate *MeAPL* genes in cassava plants.

### *MeAPL3* is critical for starch and dry matter accumulation in cassava storage roots

**Fig. 3:**
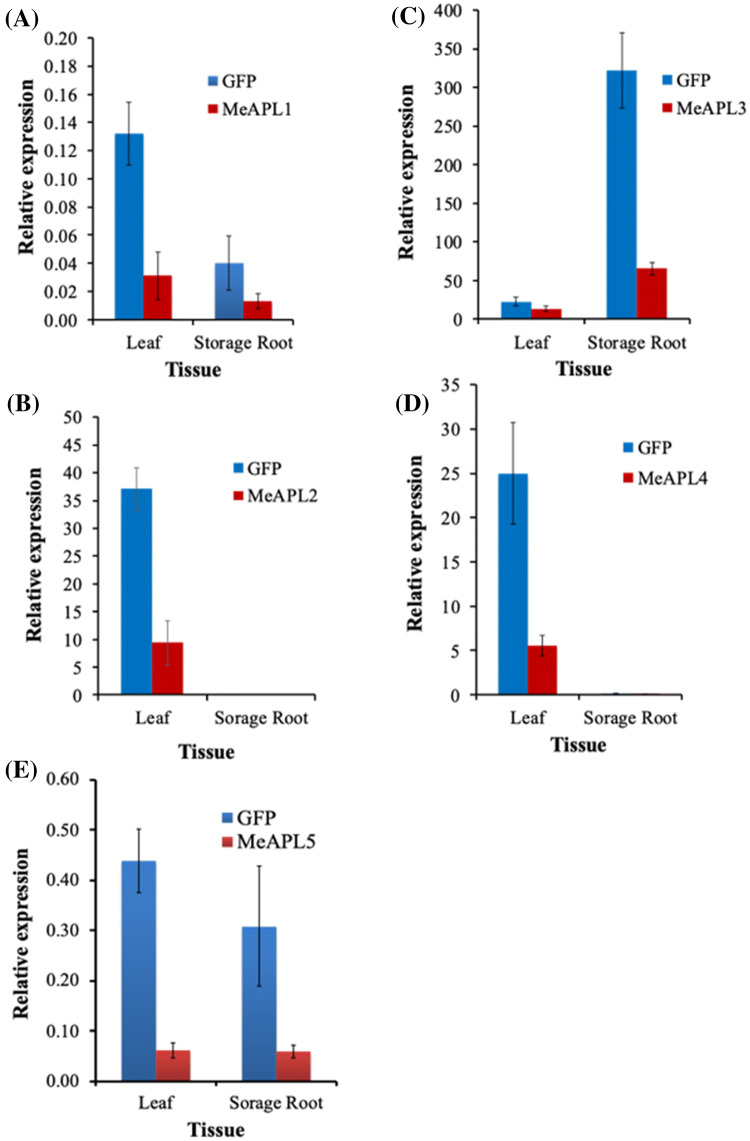
mRNA expression of cassava ADP-glucose pyrophosphorylase large subunit genes in VIGS challenged plants. Expression of **a** *MeAPL1*, **b** *MeAPL2*, **c** *MeAPL3*, **d** *MeAPL4* and **e** *MeAPL5* genes was determined by qRT-PCR in leaves and storage roots of TME 7S plants challenged with the respective VIGS-vectors for silencing and GFP-VIGS as control. Samples of storage roots and leaves were collected at harvest 12 weeks post-innoculation. Bars show SD of three biological replicates. *Note* graphs are not to the same scale

In order to determine the relative importance of each *MeAPL* gene in starch biosynthesis, the five *MeAPL* genes (*MeAPL1-MeAPL5*) were individually targeted for silencing using VIGS. VIGS constructs were generated to target each of the five family members. Four-six-week-old wild-type plants of the CMD susceptible cultivar TME 7S were inoculated with the respective VIGS vectors and grown in the greenhouse for an additional 12 weeks, after which the storage roots were harvested and assessed for DMC, starch and soluble sugar concentrations. CMD symptoms became apparent on leaves 7–15 days after inoculation. These remained mild to moderate (score 1.5–3.0 on a scale of 0–5) on all plants and did not compromise plant growth or development (Beyene et al. 2017b). To confirm that *MeAPL* genes were downregulated by VIGS, RT-qPCR analysis was performed for each of the *MeAPL* target genes and compared with the GFP-VIGS control in storage roots and leaf tissues 12 weeks after inoculation. Data presented shows a 40–78% reduction of expression in leaves for all five targeted *MeAPL* genes in inoculated plants compared to inoculated GFP-VIGS controls. In storage roots expression of *MeAPL1, MeAPL3* and *MeAPL5* was reduced by 66%, 80% and 81%, respectively (Fig. 3a–e). Expression of *MeAPL2* and *MeAPL4* was not detected in cassava storage roots by RT-qPCR assays, and was therefore consistent with data extracted from the cassava atlas (Wilson et al. 2017) (Fig. 1c).

**Fig. 4:**
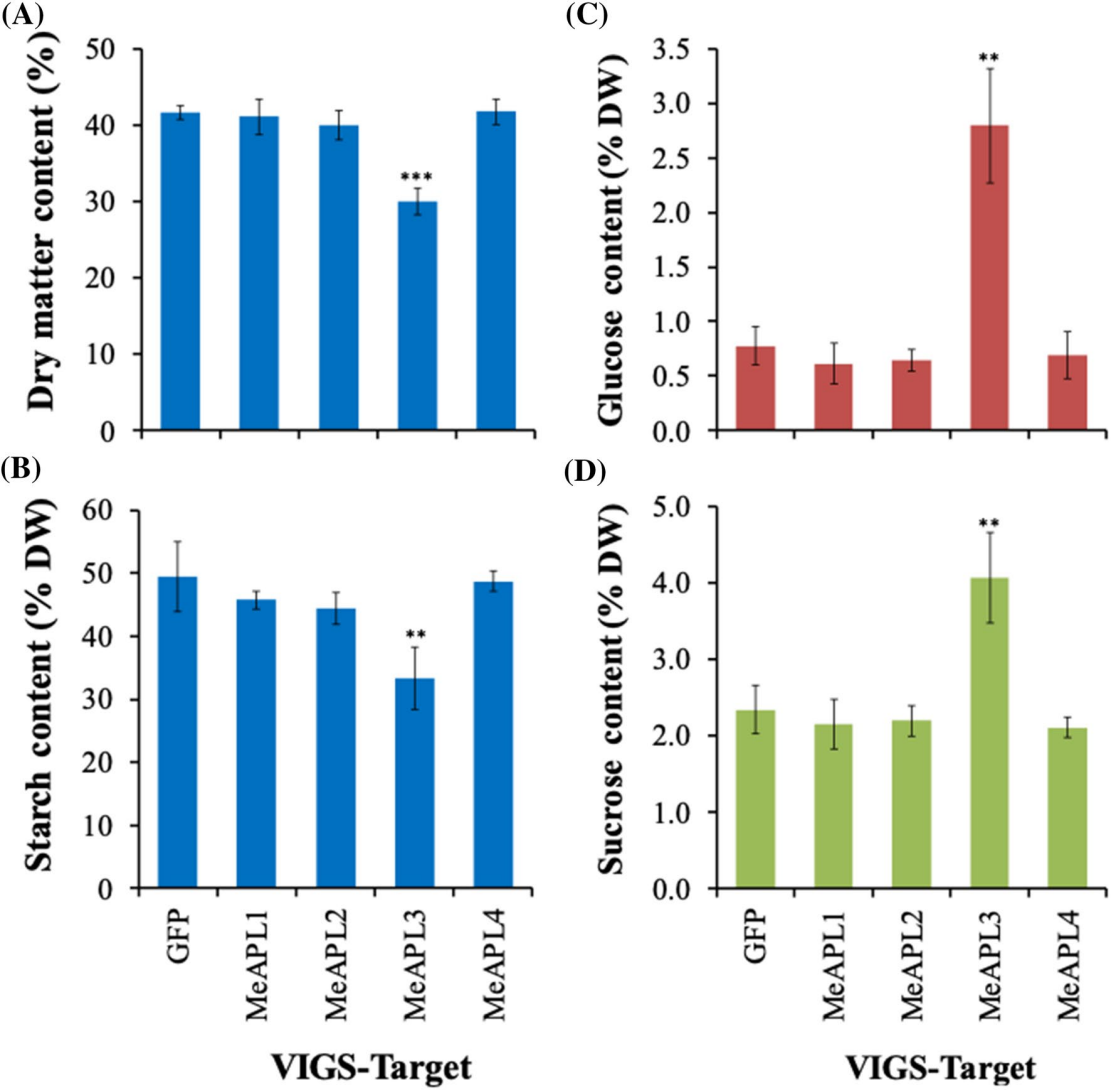
Determination of the relative importance of cassava ADP-glucose pyrophosphorylase large subunit genes in DMC and carbohydrate accumulation in cassava storage roots using EACMV-K201 VIGS. **a** Dry matter, **b** starch, **c** glucose and **d** sucrose content of cassava storage roots after challenge with VIGS vectors targeting *MeAPL1*, *MeAPL2*, *MeAPL3*, *MeAPL4* genes with GFP as control. CMD susceptible plants of cassava variety TME 7S were challenged with VIGS vectors and plants harvested 10 weeks later. Bars are SD of five biological replicates per line; ** and *** stand for significant difference, respectively, at *p* ≤ 0.01 and *p* ≤ 0.001. Student's *t*‐test compared to the GFP-VIGS control

Storage roots harvested from VIGS inoculated plants were analyzed for DMC, starch and free sugars. A significant (*p* < *0.001*) reduction from 41.6% to 30% (28% reduction) in DMC was observed in storage roots of MeAPL3-VIGS plants compared with the GFP-VIGS control (Fig. 4a). Likewise, starch content in storage roots infected with MeAPL3-VIGS was significantly (*p* < *0.01*) reduced by 33% (Fig. 4b), and soluble sugars, glucose and sucrose (Fig. 4c, d) significantly (*p* < *0.01*) elevated by 200–300% compared to GFP-VIGS controls. No significant differences were observed for DMC, starch or soluble sugars between the GFP-VIGS control and plants infected with the other three MeAPL-VIGS clones (Fig. 4a–d). Similarly, no significant differences were observed for DMC and starch content in plants inoculated with MeAPL5-VIGS and GFP-VIGS control, although soluble sugars were slightly elevated in MeAPL5-VIGS infected plants (Supplementary Fig. 1). This data indicates that *MeAPL3* is the predominant *MeAPL* gene involved in starch biosynthesis in cassava storage roots.

### Transgenic co-suppression lines of *MeAPL3* have increased storage root number and fresh weight

Transgenic plants were generated in which *MeAPL3* was co-suppressed by constitutively overexpressing the *MeAPL3* coding sequence driven by the duplicated CaMV 35S promoter. A total of 26 independent transgenic lines were recovered and confirmed to be PCR positive for presence of the *MeAPL3* transgene (data not shown). Additionally, five transgenic lines were recovered using the empty vector construct p8384. Nine *MeAPL3* transgenic lines, plus empty-vector and non-transgenic wild-type TME 7S control plants were established in the greenhouse. RT-qPCR analysis indicated that most p8388 lines selected for characterization in greenhouse showed suppression of *MeAPL3* transgene and endogenous *MeAPL3* mRNA expression, indicating characteristics of co-suppression, also known as posttranscriptional gene silencing or RNA interference (RNAi) (Supplementary Fig. 2). Six p8388 lines showed significant reduction in endogenous *MeAPL3* mRNA expression, while two lines expressed endogenous *MeAPL3*. Three transgenic lines 8388-01, 8388-2 and 8388-4 showed expression of transgene-derived *MeAPL3*, and accumulation of *MeAPL3* derived siRNA (Supplementary Fig. 2C). Transgenic lines 8388-06 and 8388-09 did not show expression of the *MeAPL3* transgene, endogenous *MeAPL3* or siRNA expression derived from these sequences. Expression of endogenous *MeAPL3* was not affected in plants transgenic for the empty vector control and was comparable to that in wild-type TME 7 (Supplementary Fig. 2).

**Fig. 5:**
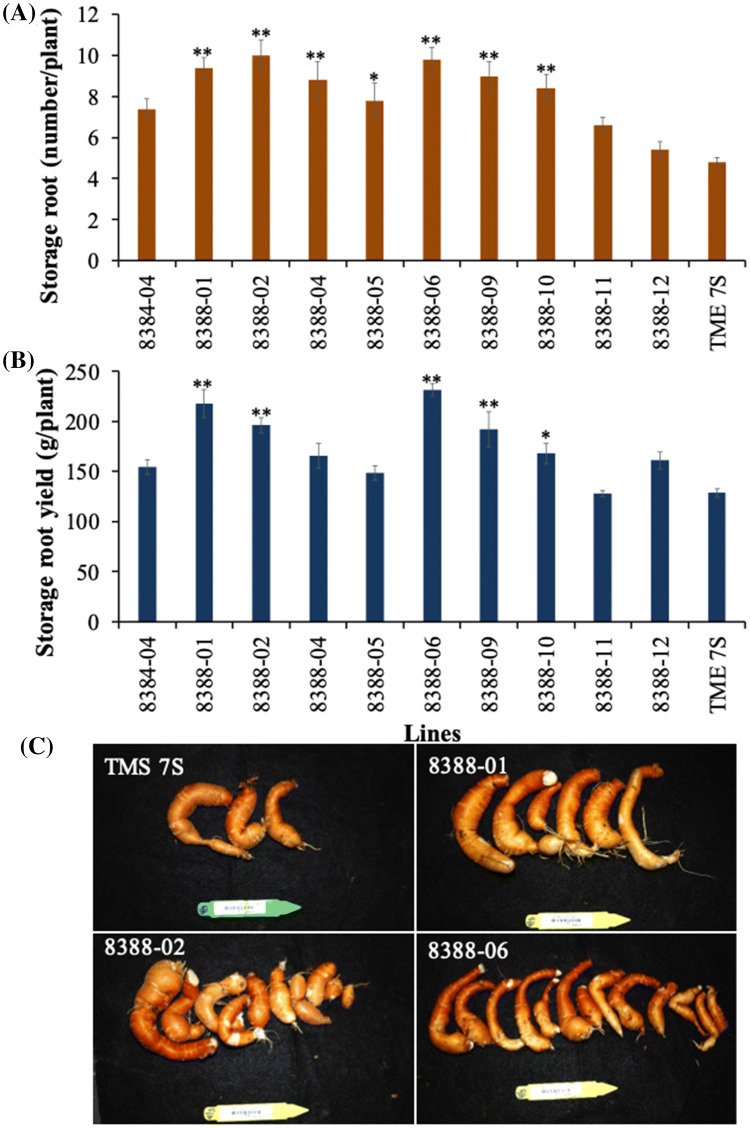
Effect of silencing of cassava *MeAPL3* on storage root production. **a** Cassava storage root number, **b** storage root yield, **c** images of storage roots from different transgenic lines, empty-vector and wild-type TME 7S controls. Transgenic lines were generated using p8384 an empty vector control (p8384) and p8388 that expresses *MeAPL3* (sense). Plant were harvested 17 weeks after planting in the greenhouse, assessed for storage root number and yield. Bars are SD of five biological replicates per line; * and ** stand for significant difference, respectively, at *p* ≤ 0.05 and *p* ≤ 0.01. Student's *t*‐test compared to the wild-type TME 7S control

After 17 weeks growth in the greenhouse *MeAPL3* co-suppression plant lines were harvested and storage roots evaluated. Six transgenic lines (8388-01, 8388-02, 8388-04, 8388-06, 8388-09, 8388-10) showed a significant (*p* < *0.01*) increase in average storage root number per plant at 8.4–10.0 compared to 5.0–7.0 in the empty vector and wild-type controls (Fig. 5a, c). Average fresh weight of storage roots per plant was elevated in *MeAPL3* co-suppressed transgenic plants reaching 168–231 g/plant, compared to wild-type controls at 127–148 g/plant (Fig. 5b).

### Transgenic co-suppression lines of *MeAPL3* have reduced starch, dry matter content and increased soluble sugars and modified leaf angle

**Fig. 6:**
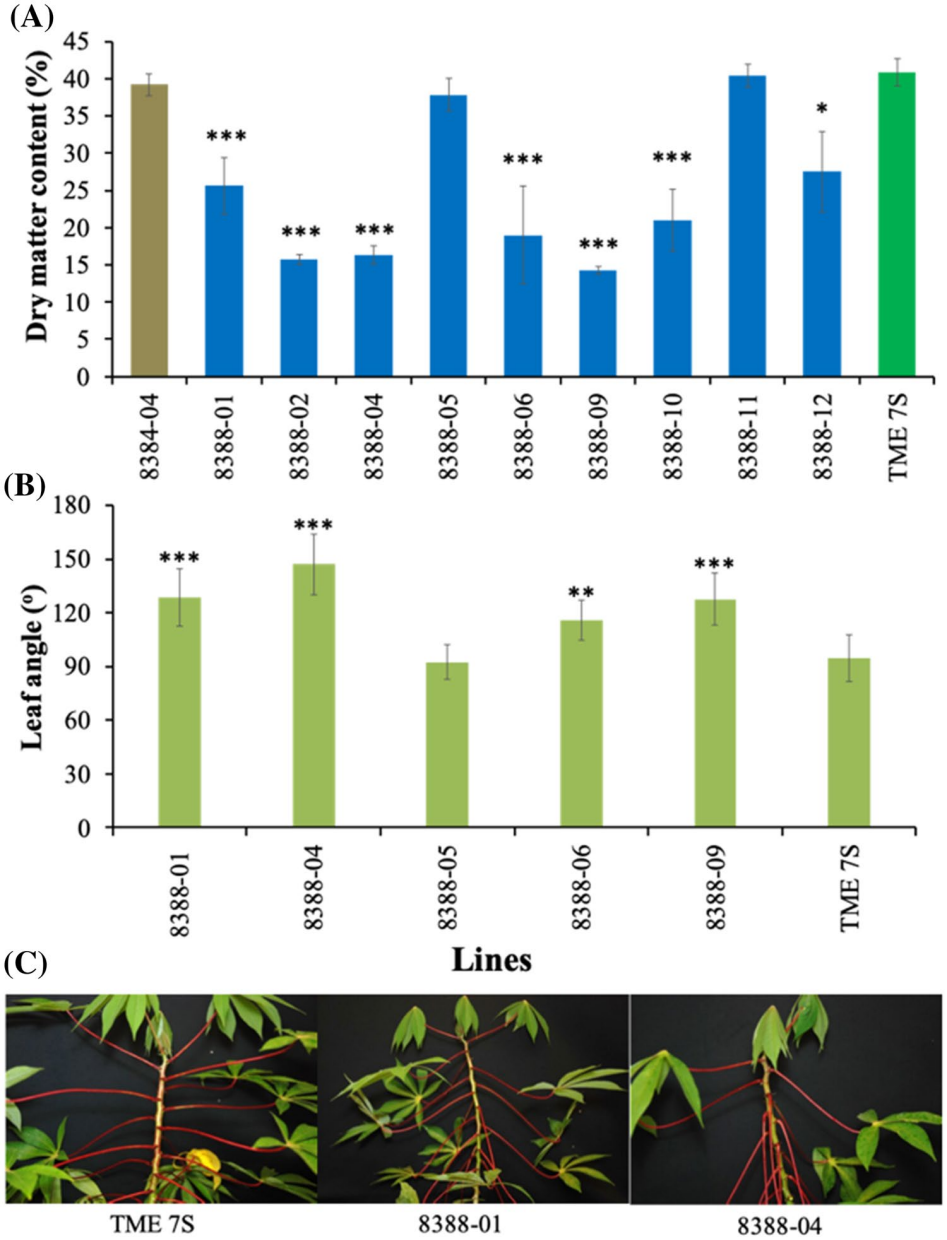
Effect of silencing *MeAPL3* on DMC and plant phenotype. **a** DMC **b** leaf angle in selected transgenic lines and wild-type control **c** plant shows partial petiole drooping of lower leaves in transgenic line 8388-01 and severe drooping in 8388-04 compared to petiole angles of wild-type TME 7S. Transgenic lines were generated using an empty vector control (p8384) and p8388 that express *MeAPL3* (sense). Plants were harvested from the greenhouse and assessed for DMC 17 weeks after planting. Average petiole angle of the middle canopy per plant was determined 10 weeks after planting by measuring leaf angle from position 8th, 9th and 10th counted downwards from the top-most leaf. Four plants per line was evaluated. Bars in (**a**) show SD of five biological replicates and Bars in (**b**) show SD of four biological replicates per lines. *, ** and *** stand for significant difference, respectively, at *p* ≤ 0.05, *p* ≤ 0.01 and *p* ≤ 0.001. Student's t‐test compared to the wild-type TME 7S control

**Fig. 7:**
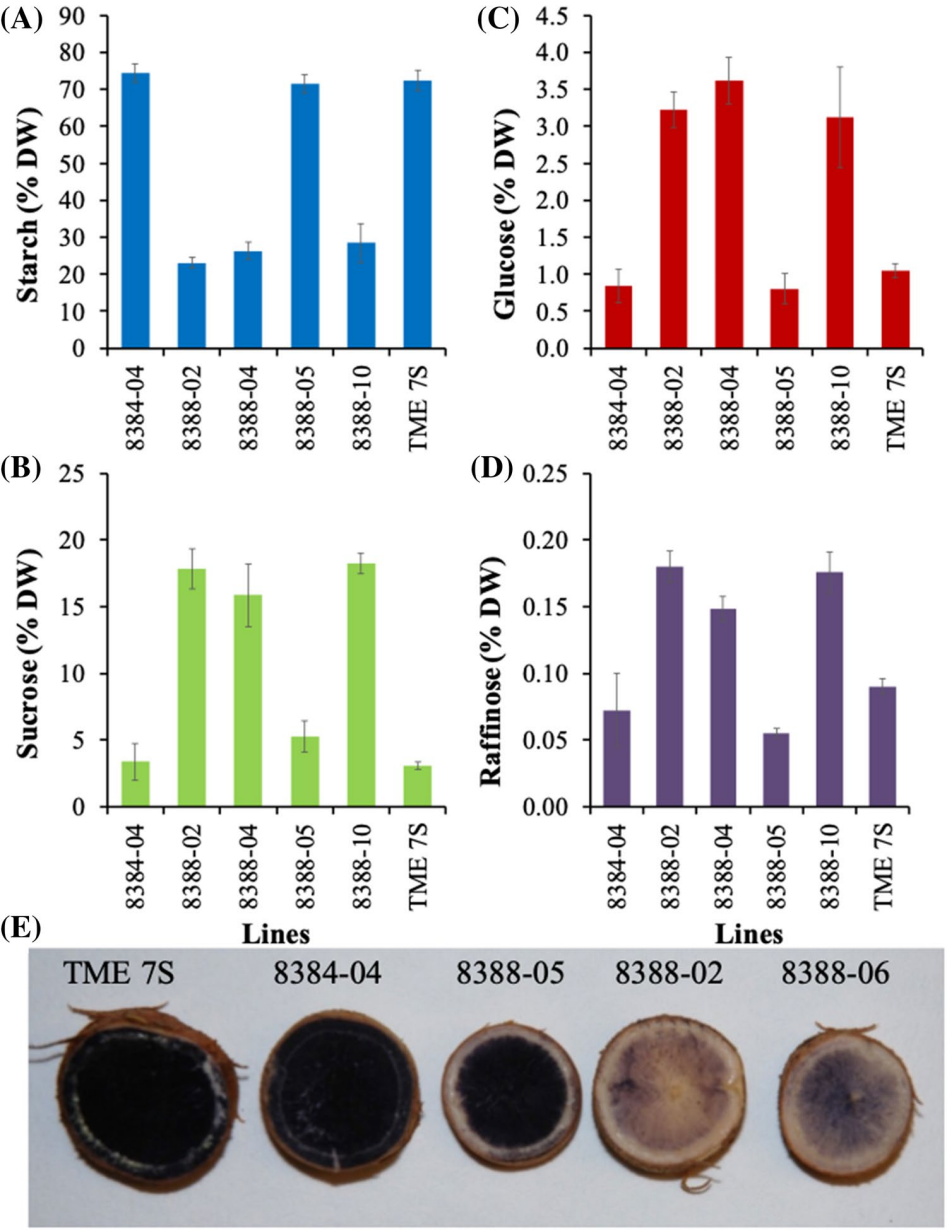
Effect of silencing *MeAPL3* on starch and soluble sugar content. **a** starch **b** sucrose, **c** glucose, **d** raffinose content and **e** starch staining of cross-sections of cassava storage roots from selected 8388 transgenic lines, empty vector control (8384) and wild-type TME 7S. Plants were harvested from the greenhouse for starch determination 17 weeks after planting as described in Fig. 5. Bars show SD of three biological replicates

At harvest 17 weeks after planting, storage roots from seven of the nine co-suppression lines showed a significant reduction in DMC, reaching only 14–27%, (a 32–65% reduction) compared to wild-type and empty vector controls at 40% DMC (Fig. 6a). A distinct phenotype was observed associated with petiole angle in *MeAPL3* co-suppressed transgenic plants. Petioles of control plants were held close to the horizontal. In contrast, in *MeAPL3* suppressed plants the petioles of mid- and lower-canopy leaves were held downwards. Measurement of petiole angles on four low DMC lines and two high DMC (transgenic 8388-05 and wild-type TME 7S) showed significant (*p*< 0.01) differences. For example, lines 8388-04 and 8388-09 which had DMC of 14 and 16% displayed leaf angles of 147^o^ and 127^o^, compared to wild-type controls with DMC of 40% which had a leaf angle of 95^o^ regardless of leaf position below the apex (Fig. 6b, c). In all cases the petiole/lamina junction adapted to cause the leaf surface to be held horizontally (Fig. 6c). Measurement of starch and soluble sugars on *MeAPL3* co-suppression lines 8388-02, 8388-04 and 8388-10 showed highly significant reduction in starch content of 23%, 26% and 28%, respectively, as compared to starch content of the wild-type and the empty vector controls each at 72% (Fig. 7a). This was consistent with reduction in DMC observed in these lines (Fig. 6a). Storage root glucose content was elevated by 200%, sucrose by 400–500% and raffinose by 65–100% in these lines compared to controls (Fig. 7c, d). Staining of storage root sections with Lugol’s IKI solution confirmed reductions in starch content, with controls staining noticeably darker than *MeAPL3* co-suppressed lines (Fig. 7e).

### *MeAPL3* co-suppression lines have reduced PPD

**Fig. 8:**
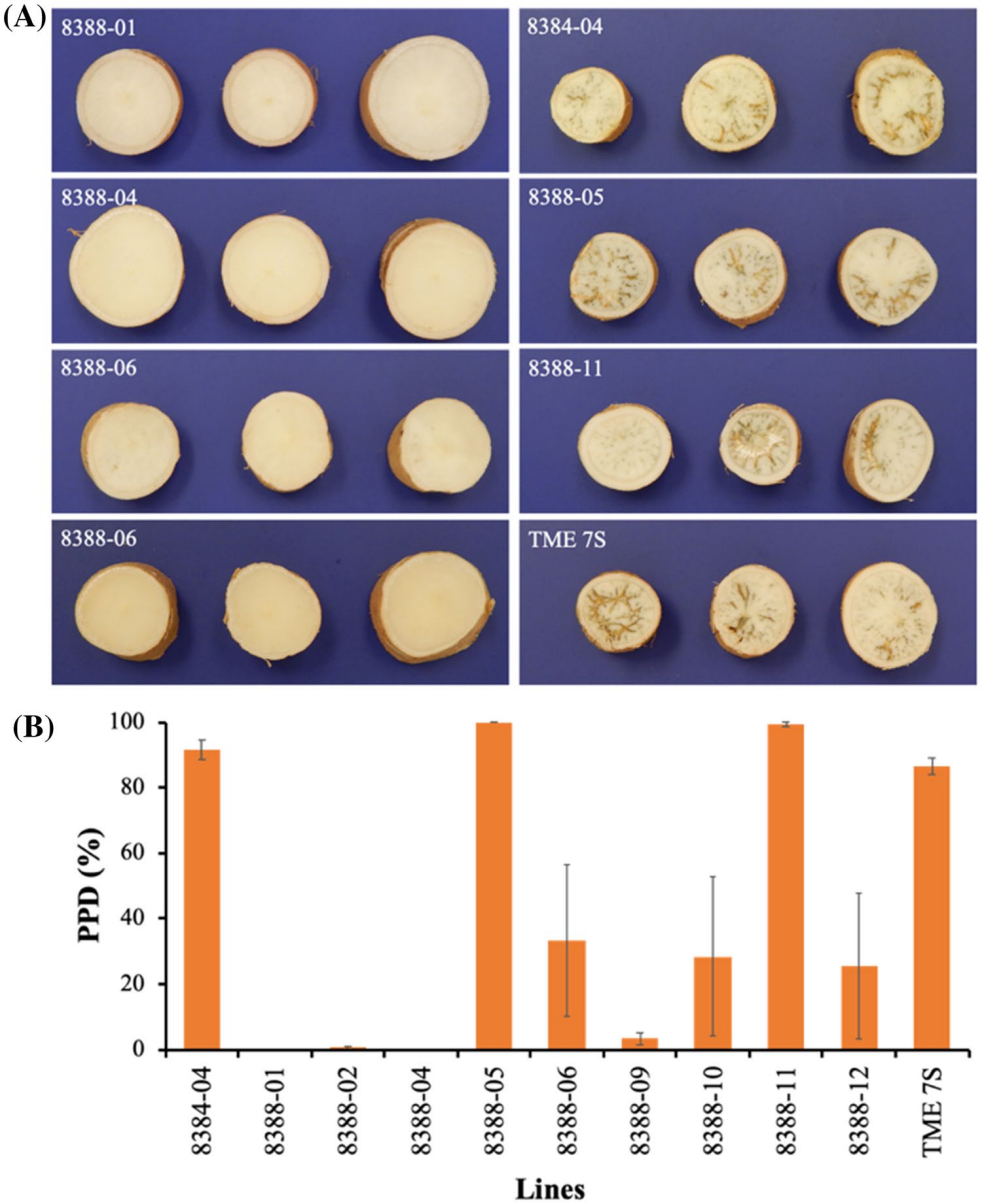
Effect of silencing *MeAPL3* on cassava postharvest physiological deterioration (PPD). **a** transverse slices of storage roots of transgenic lines showing low or no PPD (left panel) and transgenic and wild-type with high PPD (right panel) **b** PPD score of storage roots. Transgenic lines were generated using an empty vector control (p8384) and p8388 that expresses *MeAPL3* (sense). Storage roots were harvested from the greenhouse 17 weeks after planting, washed and dried with paper towels, and stored at 25 ^o^C in paper bags for three days. Roots cut transversely at proximal, middle and distal ends for PPD evaluation. PPD at day 0 is not shown as no PPD was detected at that time. Bars are SD of three biological replicates

Storage root PPD was assessed in MeAPL3-co-supression and control lines after three days of storage at 25 °C and 60% RH. Visual scoring for PPD was carried out as described by Salcedo and Siritunga (2011) on transverse slices obtained from proximal, middle and distal portions of the storage root. Data indicate that lines with reduced starch and DMC had no, or very low, incidence of PPD, while transgenic lines with high starch and DMC had very high PPD incidence (Fig. 8). PPD incidence was significantly (*p* < *0.01*) higher at 87–100% in high DMC/high starch lines including wild-type TME 7S and transgenic lines 8384 (empty vector control), 8388-05 and 8388-11. In contrast, PPD incidence was lower at 25–33% in lines 8388-06, 8388-10 and 8388-12 and very low at 0–3% in the remaining three transgenic *MeAPL3* co-suppression lines at the end of the three day assessment period (Fig. 8).

## Discussion

In this study, functional characterization of five *MeAPL* genes that code for the large subunit of ADP-glucose pyrophosphorylase is presented, showing their preferential expression in cassava leaves and storage roots. Silencing each of these genes individually using VIGS showed that starch synthesis and accumulation in cassava storage roots is facilitated very largely by *MeAPL3*. This isoform is by far the most abundantly expressed isoform in cassava storage roots, fibrous roots and stems compared to the other *MeAPL* isoforms identified in this study. By generating stable transgenic co-suppression lines of *MeAPL3*, we showed that DMC and starch were significantly reduced (by up to 65%), thereby substantiating data generated by VIGS. We also demonstrated that transgenic cassava lines with reduced starch and DMC had low incidence of PPD, showing a strong, direct relationship between starch, DMC and incidence of PPD. We also further showed that transgenic *MeAPL3* co-suppression lines with reduced starch and DMC presented a droopy leaf phenotype characterized by increased leaf angle.

Dong et al. (2019) recently described gene structures of cassava AGPase genes that included four large and three small subunits and their conserved domains. The *MeAPL3* identified and characterized in the present study is most likely the same as cassava *shrunken-2* (*sh2*) (Munyikwa et al. 2001), the only differences being (1) the deduced amino acid sequence described here is 527 aa residues compared to 531 by Munyikwa et al. (2001) or 528 aa by Dong et al. (2019), and (2) abundance of expression of this gene is higher in storage roots than in leaves, as shown in Fig. 3c and expression data obtained from cassava Atlas (Fig. 1c) (Wilson et al. 2017). We identified at least five loci that code for *MeAPL* genes in the v6.1 cassava genome. Four of these genes had full gene structure and ORF and are annotated as such (Dong et al. 2019). The *MeAPL5* (GenBank MN734216) identified here is represented by two partial, but overlapping sequences, one on chromosome 18 with the remaining partial sequence on an un-anchored scaffold (Manes.S107700). This could be due to assembly related errors in the cassava reference genome.

Multiple *APL* genes have been reported from different plant species, four in rice (Lee et al. 2007), four in corn (Huang et al. 2014), three in potato (Van Harsselaar et al. 2017) and four in Arabidopsis (Crevillén et al. 2003, 2005). Here, we show presence of not four as reported by Dong et al. (2019), but five isoforms of *APL* genes in cassava (Fig. 1). Different isoforms of *APL* genes are known to be expressed preferentially in different tissues and organs. For example, in rice *APL2* and *APS2b* are endosperm specific, while *APS2a* and *APL3* are expressed preferentially in leaves (Hirose et al. 2006; Lee et al. 2007). Similarly, in *A. thaliana APL1* and *APS1* are preferentially expressed in photosynthetic (source) tissues while *APL3* and *APL4* are expressed preferentially in roots and sugar-inducible in leaves (Crevillén et al. 2005; Streb and Zeeman 2012). In heterotrophic tissue of potato tubers APL3 (StAPL1 in this study, Fig. 1a and b) is preferentially expressed, while APL1 (StAPL3 in this study, Fig. 1a and b) is expressed mainly in leaves (Van Harsselaar et al. 2017). Expression assays in leaves and storage roots of VIGS-challenged plants (Fig. 3) coupled with data from the cassava transcriptome Atlas (Wilson et al. 2017) (Fig. 1c) clearly show preferential expression of the different *MeAPL* isoforms in different organs. Specifically, isoform *MeAPL3* is abundantly expressed in storage roots, fibrous roots and stems (Figs. 1c and 3c). In leaves, the closely related *MeAPL2* and *MeAPL4* (89.85% identity at amino acid level) are expressed at higher levels than *MeAPL3*. Tissue specific expression of different AGPase genes is suggested to be due to functional specialization in source and sink tissue, and as such, might have different sensitivity to allosteric regulations (Ballicora et al. 2005; Nakata and Okita 1995). The *MeAPL3* characterized in this study codes for conserved amino acid Q and T at position 102 and 112, respectively of the catalytic region (Fig. 1a) in a manner consistent with group III large subunit from different plant species that preferentially express in the tissues of sink organs such as tubers, stem and roots (Ballicora et al. 2005). In contrast, *MeAPL1*, *MeAPL2*, *MeAPL4* and *MeAPL5* have conserved residues amino acids R and K at position 102 and 112, respectively within the catalytic region common to group I and group II types based on phylogenetic classification by Ballicora et al. (2005). These genes tend to express in source tissues.

By presenting evidence from transient studies using VIGS and multiple stable co-suppression plants lines we showed that *MeAPL3* is the major cassava *APL* isoform involved in starch accumulation in storage roots (Fig. 4, Supplementary Fig. 1, Figs. 6 and 7). This is consistent with other studies of *shrunken-2* in maize (Bhave et al. 1990), rice *APL2* (Lee et al. 2007), and RNAi suppression lines (Xu et al. 2019). An interesting associated observation was an increase in average storage root number per plant in lines with reduced starch and DMC. Transgenic potato expressing antisense cassava (Munyikwa et al. 2001) and potato AGPase small subunits (Müller-Röber et al. 1992) also displayed increased tuber number, tuber fresh weight and reduction in tuber DMC.

In the current study, we demonstrated that silencing *MeAPL3* using VIGS resulted in approximately 30% reduction in storage root DMC and starch content (Fig. 4). Transgenic *MeAPL3* co-suppression lines showing reduced expression of *MeAPL3* (Supplementary Fig. 2) also had significantly reduced starch and DMC (Figs. 6 and 7). Lines with reduced DMC and starch content displayed increases in soluble sugars (Figs. 4 and 7), a phenotype that we previously reported in transgenic cassava lines accumulating carotenoids (Beyene et al. 2018). These characteristics have also been reported in transgenic RNAi pea lines (Weigelt et al. 2009), and in transgenic potato expressing an antisense of potato small subunit of AGPase that resulted in increased soluble sugars and reduction in tuber DMC (Müller-Röber et al. 1992).

Leaf angle is an important plant trait that determines light interception and productivity (Drewry et al. 2014; Truong et al. 2015). Transgenic cassava lines with significantly reduced DMC and starch content displayed a unique droopy leaf phenotype on the middle and lower canopies. On the same plants newly formed, fully expanded leaves occupying the upper quarter of the canopy grew as wild-type (Fig. 6b, c). This change in petiole/stem angle has not been reported in cassava, but correlates with studies describing droopy leaf phenotype associated with mutation of droppy leaf (*drl*) genes in *Zea mays* (Strable et al. 2017). The mechanism of how starch content relates to leaf angle is not clear, but it is possible that starch at the stem/petiole junction might play a role as shown previously in which amyloplast displacement (starch content) affected gravitropism (Weise et al. 2000; Morita and Tasaka 2004). Further investigations are needed to determine the molecular basis and physiological mechanism of this phenotype.

A predominant problem in production and utilization of cassava is the very short shelf-life of harvested storage roots, with 20–30% of yields estimated to be lost due to PPD every year (Djabou et al. 2017; Zainuddin et al. 2018). This limits the market potential and value of the crop across all its growing regions. Previous studies have implicated, but not proven, a positive correlation between high DMC and severity of PPD in cassava storage roots (Sánchez et al. 2006). We previously reported that transgenic cassava lines accumulating carotenoids have significantly reduced PPD (Beyene et al. 2018). In addition to reduced DMC, these plants were characterized by elevated levels of soluble sugars, triacylglycerols and total fatty acids, and reduced starch content (Beyene et al. 2018), making it problematic to determine what might be influencing reduced severity of PPD. Conventionally bred orange-fleshed cassava varieties also have low or no PPD incidences (Sánchez et al. 2006; Morante et al. 2010). Previous reports were inconclusive as to whether the reduction of PPD incidence was due to altered metabolite changes including reduced DMC and starch, or the direct effect of carotenoid accumulation on PPD.

Harvested cassava roots accumulate elevated levels of reactive oxygen species (ROS) (Reilly et al. 2003; Iyer et al. 2010; Zidenga et al. 2012). Reduced PPD in high beta-carotene accumulating cassava storage roots has been suggested to be due to the anti-oxidant properties of carotenoids (Morante et al. 2010; Zidenga et al. 2012). In line with this hypothesis, transgenic elevation of APX (Vanderschuren et al. 2014) and alternative oxidase AOX (Zidenga et al. 2012) and co-expression of copper/zinc superoxide dismutase (Xu et al. 2013) reduced PPD due to the antioxidant properties of these enzymes. In the present study, a strong relationship is shown between incidence of PPD and dry matter/starch content, where lines with high dry matter and starch content showed 70–100% PPD after three days of storage, while lines with reduced DMC and starch content had reduced or no incidence of PPD. This clearly demonstrates PPD incidence and severity to be tightly related with storage root dry matter/starch content. Although previous studies have implicated the existence of a relationship between DMC and PPD, these relied on obsevations across different genotypes with different genetic backgrounds (Van Oirschot et al. 2000; Sánchez et al. 2006; Morante et al. 2010) or treatments like pruning (Van Oirschot et al. 2000). Sugars are known for their antioxidant properties, either by serving as signaling molecules that trigger gene expression involved in ROS scavenging (Couée et al. 2006) or directly where high concentration of sugars can function as ROS scavengers (Van Den Ende and Valluru 2009; Bolouri-Moghaddam et al. 2010). Our study shows that silencing of the *MeAPL3* increases the pool of soluble sugars (both reducing and non-reducing). It remains unknown whether these sugars are critical in perturbing the incidence of PPD observed in this study or in transgenic and conventionally bred orange-fleshed cassava.

In conclusion, *MeAPL3* has been identified as the main isoform of the large subunit genes reponsible for starch biosynthesis in cassava storage roots. We also show a direct relationship between storage root DMC/starch content and cassava PPD. Open questions remain, concerning the molecular and biochemical bases linking high starch to enhanced PPD and how low starch leads to droopy phenotype in leaves.

## Electronic supplementary material

Below is the link to the electronic supplementary material.

Supplementary Figure 1. Determination of the relative importance of cassava *MeAPL5* in DMC and carbohydrate accumulation in cassava storage roots using EACMV-K201 VIGS. (A) Dry matter, (B) starch, (C) glucose and (D) sucrose content of cassava storage roots after challenge with VIGS vectors targeting *MeAPL5*, with GFP as a control. CMD susceptible plants of cassava cultivar TME 7S were challenged with VIGS vectors and harvested five weeks later. A slight increase in soluble sugars was observed, with no significant differences in starch and DMC in storage roots between GFP-VIGS and MeAPL5-VIGS treated plants. Bars are SD of five biological replicates per line; * stands for significant difference, respectively, at *P* ≤ 0.05. Student's *t*‐test compared to the GFP-VIGS control. (TIFF 2028 kb)

Supplementary Figure 2. mRNA and sRNA expression of transgene and endogenous *MeAPL3* in transgenic cassava *MeAPL3* co-suppression lines. Expression of (A) endogenous *MeAPL3*, (B) transgenic *MeAPL3*, (C) siRNA derived from *MeAPL3* in leaves. Transgenic lines were generated using an empty vector control (p8384) and p8388 that express MeAPL3 (sense). Leaf samples for assay were collected 17 weeks after planting in the greenhouse from the third fully expanded leaf for mRNA and sRNA extraction. sRNA blots was generated (Beyene et al., 2017a), using sRNA probes generated from cloned full-length *MeAPL3*. (TIFF 2028 kb)

Supplementary Table 1. Primers used for cloning, PCR and RT-qPCR assay of different genes. (PDF 69 kb)

## References

[CR1] AACC (2000). Approved methods of the American association of cereal chemists.

[CR3] Ballicora MA, Fu Y, Nesbitt NM, Preiss J (1998). ADP-glucose pyrophosphorylase from potato tubers. Site-directed mutagenesis studies of the regulatory sites. Plant Physiol.

[CR4] Ballicora MA, Iglesias AA, Preiss J (2004). ADP-glucose pyrophosphorylase: a regulatory enzyme for plant starch synthesis. Photosynth Res.

[CR2] Ballicora MA, Dubay JR, Devillers CH, Preiss J (2005). Resurrecting the ancestral enzymatic role of a modulatory subunit. J Biol Chem.

[CR5] Bart RS, Taylor NJ (2017). New opportunities and challenges to engineer disease resistance in cassava, a staple food of African small-holder farmers. PLoS Pathog.

[CR6] Beeching JR, Han Y, Gómez-Vásquez R, Day RC, Cooper RM (1998). Wound and defense responses in cassava as related to post-harvest physiological deterioration. Phytochem Signals Plant Microbe Interact.

[CR9] Beyene G, Chauhan RD, Wagaba H, Moll T, Alicai T, Miano D (2016). Loss of CMD2-mediated resistance to cassava mosaic disease in plants regenerated through somatic embryogenesis. Mol Plant Pathol.

[CR7] Beyene G, Chauhan RD, Ilyas M, Wagaba H, Fauquet CM, Miano D (2017). A virus-derived stacked RNAi construct confers robust resistance to cassava brown streak disease. Front Plant Sci.

[CR8] Beyene G, Chauhan RD, Taylor NJ (2017). A rapid virus-induced gene silencing (VIGS) method for assessing resistance and susceptibility to cassava mosaic disease. Virol J.

[CR10] Beyene G, Solomon FR, Chauhan RD, Gaitán-Solis E, Narayanan N, Gehan J (2018). Provitamin A biofortification of cassava enhances shelf life but reduces dry matter content of storage roots due to altered carbon partitioning into starch. Plant Biotechnol J.

[CR11] Bhave MR, Lawrence S, Barton C, Hannah LC (1990). ldentification and molecular characterization of. Science.

[CR12] Bolouri-Moghaddam MR, Le Roy K, Xiang L, Rolland F, Van Den Ende W (2010). Sugar signalling and antioxidant network connections in plant cells. FEBS J.

[CR13] Booth RH (1976). Storage of fresh cassava (*Manihot esculenta*). I. Post-harvest deterioration and its control. Exp Agric.

[CR14] Bredeson JV, Lyons JB, Prochnik SE, Wu GA, Ha CM, Edsinger-Gonzales E (2016). Sequencing wild and cultivated cassava and related species reveals extensive interspecific hybridization and genetic diversity. Nat Biotechnol.

[CR15] Bull SE, Ndunguru J, Gruissem W, Beeching JR, Vanderschuren H (2011). Cassava: constraints to production and the transfer of biotechnology to African laboratories. Plant Cell Rep.

[CR16] Bull SE, Seung D, Chanez C, Mehta D, Kuon JE, Truernit E (2018). Accelerated ex situ breeding of GBSS- and PTST1-edited cassava for modified starch. Sci Adv.

[CR17] Chauhan RD, Beyene G, Kalyaeva M, Fauquet CM, Taylor N (2015). Improvements in Agrobacterium-mediated transformation of cassava (*Manihot esculenta* Crantz) for large-scale production of transgenic plants. Plant Cell Tissue Organ Cult.

[CR18] Copeland L, Preiss J (1981). Purification of spinach leaf ADPglucose pyrophosphorylase. Plant Physiol.

[CR19] Couée I, Sulmon C, Gouesbet G, El Amrani A (2006). Involvement of soluble sugars in reactive oxygen species balance and responses to oxidative stress in plants. J Exp Bot.

[CR20] Crevillén P, Ballicora MA, Mérida Á, Preiss J, Romero JM (2003). The different large subunit isoforms of *Arabidopsis thaliana* ADP-glucose pyrophosphorylase confer distinct kinetic and regulatory properties to the heterotetrameric enzyme. J Biol Chem.

[CR21] Crevillén P, Ventriglia T, Pinto F, Orea A, Mérida Á, Romero JM (2005). Differential pattern of expression and sugar regulation of *Arabidopsis thaliana* ADP-glucose pyrophosphorylase-encoding genes. J Biol Chem.

[CR22] Djabou ASM, Carvalho LJCB, Li QX, Niemenak N, Chen S (2017). Cassava postharvest physiological deterioration: a complex phenomenon involving calcium signaling, reactive oxygen species and programmed cell death. Acta Physiol Plant.

[CR23] Dong M-Y, Fan X-W, Li Y-Z (2019). Cassava AGPase genes and their encoded proteins are different from those of other plants. Planta.

[CR24] Doyle JJ, Doyle J (1990). Islotaion of plant DNA from fresh tissue. Focus (Madison).

[CR25] Drewry DT, Kumar P, Long SP (2014). Simultaneous improvement in productivity, water use, and albedo through crop structural modification. Glob Chang Biol.

[CR26] Geigenberger P (2011). Regulation of starch biosynthesis in response to a fluctuating environment. Plant Physiol.

[CR27] Hirose T, Ohdan T, Nakamura Y, Terao T (2006). Expression profiling of genes related to starch synthesis in rice leaf sheaths during the heading period. Physiol Plant.

[CR28] Howeler R, Lutaladio N, Thomas G (2013). Save and grow: cassava. A guide to sustainable production intensificationProduire plus avec moins Ahorrar para crecer.

[CR29] Huang B, Hennen-Bierwagen TA, Myers AM (2014). Functions of multiple genes encoding ADP-glucose pyrophosphorylase subunits in maize endosperm, embryo, and leaf. Plant Physiol.

[CR30] Iyer S, Mattinson DS, Fellman JK (2010). Study of the early events leading to cassava root postharvest deterioration. Trop Plant Biol.

[CR31] Kakehi K, Honda S, Biermann CJ, McGinnis GD (1989). Silyl ethers of carbohydrates. Analysis of carbohydrates by GLC and MS.

[CR32] Kay R, Chan AMY, Daly M, McPherson J (1987). Duplication of CaMV 35S promoter sequences creates a strong enhancer for plant genes. Science.

[CR33] Lee SK, Hwang SK, Han M, Eom JS, Kang HG, Han Y (2007). Identification of the ADP-glucose pyrophosphorylase isoforms essential for starch synthesis in the leaf and seed endosperm of rice (*Oryza sativa* L.). Plant Mol Biol.

[CR34] Marchler-Bauer A, Derbyshire MK, Gonzales NR, Lu S, Chitsaz F, Geer LY (2014). CDD: NCBI’s conserved domain database. Nucleic Acids Res.

[CR35] Marriott J, Been BO, Perkins C (1978). The aetiology of vascular discoloration in cassava roots after harvesting: association with water loss from wounds. Physiol Plant.

[CR36] Morante N, Sánchez T, Ceballos H, Calle F, Pérez JC, Egesi C (2010). Tolerance to postharvest physiological deterioration in cassava roots. Crop Sci.

[CR37] Moreno I, Gruissem W, Vanderschuren H (2011). Reference genes for reliable potyvirus quantitation in cassava and analysis of Cassava brown streak virus load in host varieties. J Virol Methods.

[CR38] Morita MT, Tasaka M (2004). Gravity sensing and signaling. Curr Opin Plant Biol.

[CR39] Müller-Röber B, Sonnewald U, Willmitzer L (1992). Inhibition of the ADP-glucose pyrophosphorylase in transgenic potatoes leads to sugar-storing tubers and influences tuber formation and expression of tuber storage protein genes. EMBO J.

[CR40] Munyikwa TRI, Kreuze J, Fregene M, Suurs L, Jacobsen E, Visser RGF (2001). Isolation and characterisation of cDNAs encoding the large and small subunits of ADP-glucose pyrophosphorylase from cassava (*Manihot esculenta* Crantz). Euphytica.

[CR41] Nakata PA, Okita TW (1995). Differential regulation of ADP-glucose pyrophosphorylase in the sink and source tissues of potato. Plant Physiol.

[CR42] Ogwok E, Alicai T, Rey MEC, Beyene G, Taylor NJ (2015). Distribution and accumulation of cassava brown streak viruses within infected cassava (*Manihot esculenta*) plants. Plant Pathol.

[CR73] Preiss J (1984). Bacterial glycogen synthesis and its regulation. Ann Rev Microbiol.

[CR43] Raemakers K, Schreuder M, Suurs L, Furrer-Verhorst H, Vincken JP, De Vetten N (2005). Improved cassava starch by antisense inhibition of granule-bound starch synthase I. Mol Breed.

[CR45] Reilly K, Gómez-Vásquez R, Buschmann H, Tohme J, Beeching JR (2003). Oxidative stress responses during cassava post-harvest physiological deterioration. Plant Mol Biol.

[CR44] Reilly K, Bernal D, Cortés DF, Gómez-Vásquez R, Tohme J, Beeching JR (2007). Towards identifying the full set of genes expressed during cassava post-harvest physiological deterioration. Plant Mol Biol.

[CR46] Rickard JE (1985). Physiological deterioration of cassava roots. J Sci Food Agric.

[CR47] Saithong T, Rongsirikul O, Kalapanulak S, Chiewchankaset P, Siriwat W, Netrphan S (2013). Starch biosynthesis in cassava: A genome-based pathway reconstruction and its exploitation in data integration. BMC Syst Biol.

[CR48] Salcedo A, Siritunga D (2011). Insights into the physiological, biochemical and molecular basis of postharvest deterioration in cassava (*Manihot esculenta*) roots. Am J Exp Agric.

[CR49] Sánchez T, Chávez AL, Ceballos H, Rodriguez-Amaya DB, Nestel P, Ishitani M (2006). Reduction or delay of post-harvest physiological deterioration in cassava roots with higher carotenoid content. J Sci Food Agric.

[CR50] Sánchez T, Salcedo E, Ceballos H, Dufour D, Mafla G, Morante N (2009). Screening of starch quality traits in cassava (*Manihot esculenta* Crantz). Starch Stärke.

[CR51] Smith AM (2008). Prospects for increasing starch and sucrose yields for bioethanol production. Plant J.

[CR74] Stark DM, Timmerman KP, Barry GF, Preiss J, Kishore GM (1992). Regulation of the amount of starch in plant tissues by ADP glucose pyrophosphorylase. Science.

[CR52] Strable J, Wallace JG, Unger-Wallace E, Briggs S, Bradbury PJ, Buckler ES (2017). Maize YABBY Genes drooping leaf1 and drooping leaf2 Regulate Plant Architecture. Plant Cell.

[CR53] Streb S, Zeeman SC (2012). Starch metabolism in arabidopsis. Arabidopsis B.

[CR54] Tappiban P, Smith DR, Triwitayakorn K, Bao J (2019). Recent understanding of starch biosynthesis in cassava for quality improvement: a review. Trends Food Sci Technol.

[CR55] Taylor N, Gaitán-Solís E, Moll T, Trauterman B, Jones T, Pranjal A (2012). A high-throughput platform for the production and analysis of transgenic cassava (*Manihot esculenta*). Plants Trop Plant Biol.

[CR56] Truong SK, McCormick RF, Rooney WL, Mullet JE (2015). Harnessing genetic variation in leaf angle to increase productivity of Sorghum bicolor. Genetics.

[CR57] Tuncel A, Kawaguchi J, Ihara Y, Matsusaka H, Nishi A, Nakamura T (2014). The rice endosperm ADP-glucose pyrophosphorylase large subunit is essential for optimal catalysis and allosteric regulation of the heterotetrameric enzyme. Plant Cell Physiol.

[CR58] Uarrota VG, Maraschin M (2015). Metabolomic, enzymatic, and histochemical analyzes of cassava roots during postharvest physiological deterioration Bioinformatics. BMC Res Notes.

[CR59] Van Den Ende W, Valluru R (2009). Sucrose, sucrosyl oligosaccharides, and oxidative stress: scavenging and salvaging?. J Exp Bot.

[CR60] Van Harsselaar JK, Lorenz J, Senning M, Sonnewald U, Sonnewald S (2017). Genome-wide analysis of starch metabolism genes in potato (*Solanum tuberosum* L.). BMC Genomics.

[CR61] Van Oirschot QEA, O’Brien GM, Dufour D, El-Sharkawy MA, Mesa E (2000). The effect of pre-harvest pruning of cassava upon root deterioration and quality characteristics. J Sci Food Agric.

[CR62] Vanderschuren H, Nyaboga E, Poon JS, Baerenfaller K, Grossmann J, Hirsch-Hoffmann M (2014). Large-scale proteomics of the cassava storage root and identification of a target gene to reduce postharvest deterioration. Plant Cell.

[CR63] Ventriglia T, Kuhn ML, Ruiz MT, Ribeiro-Pedro M, Valverde F, Ballicora MA (2008). Two Arabidopsis ADP-glucose pyrophosphorylase large subunits (APL1 and APL2) are catalytic. Plant Physiol.

[CR64] Wang W, Hostettler CE, Damberger FF, Kossmann J, Lloyd JR, Zeeman SC (2018). Modification of cassava root starch phosphorylation enhances starch functional properties. Front Plant Sci.

[CR65] Weigelt K, Küster H, Rutten T, Fait A, Fernie AR, Miersch O (2009). ADP-glucose pyrophosphorylase-deficient pea embryos reveal specific transcriptional and metabolic changes of carbon-nitrogen metabolism and stress responses. Plant Physiol.

[CR66] Weise SE, Kuznetsov OA, Hasenstein KH, Kiss JZ (2000). Curvature in Arabidopsis inflorescence stems is limited to the region of amyloplast displacement. Plant Cell Physiol.

[CR67] Wilson MC, Mutka AM, Hummel AW, Berry J, Chauhan RD, Vijayaraghavan A (2017). Gene expression atlas for the food security crop cassava. New Phytol.

[CR68] Xu J, Duan X, Yang J, Beeching JR, Zhang P (2013). Enhanced reactive oxygen species scavenging by overproduction of superoxide dismutase and catalase delays postharvest physiological deterioration of cassava storage roots. Plant Physiol.

[CR69] Xu X, Vanhercke T, Shrestha P, Luo J, Akbar S, Konik-Rose C (2019). Upregulated lipid biosynthesis at the expense of starch production in potato (*Solanum tuberosum*) vegetative tissues via simultaneous downregulation of ADP-glucose pyrophosphorylase and sugar dependent1 expressions. Front Plant Sci.

[CR70] Zainuddin IM, Fathoni A, Sudarmonowati E, Beeching JR, Gruissem W, Vanderschuren H (2018). Cassava post-harvest physiological deterioration: from triggers to symptoms. Postharvest Biol Technol.

[CR71] Zeeman SC, Kossmann J, Smith AM (2010). Starch: its metabolism, evolution, and biotechnological modification in plants. Annu Rev Plant Biol.

[CR72] Zidenga T, Leyva-Guerrero E, Moon H, Siritunga D, Sayre R (2012). Extending Cassava root shelf life via reduction of reactive oxygen species production. Plant Physiol.

