# Supplementary figures and images for: Cassava shrunken-2 homolog MeAPL3 determines storage root starch and dry matter content and modulates storage root postharvest physiological deterioration
Source: Plant Mol Biol. 2020 Oct 6;109(3):283–99. doi: 10.1007/s11103-020-00995-z (PMC9163024; doi:10.1007/s11103-020-00995-z)

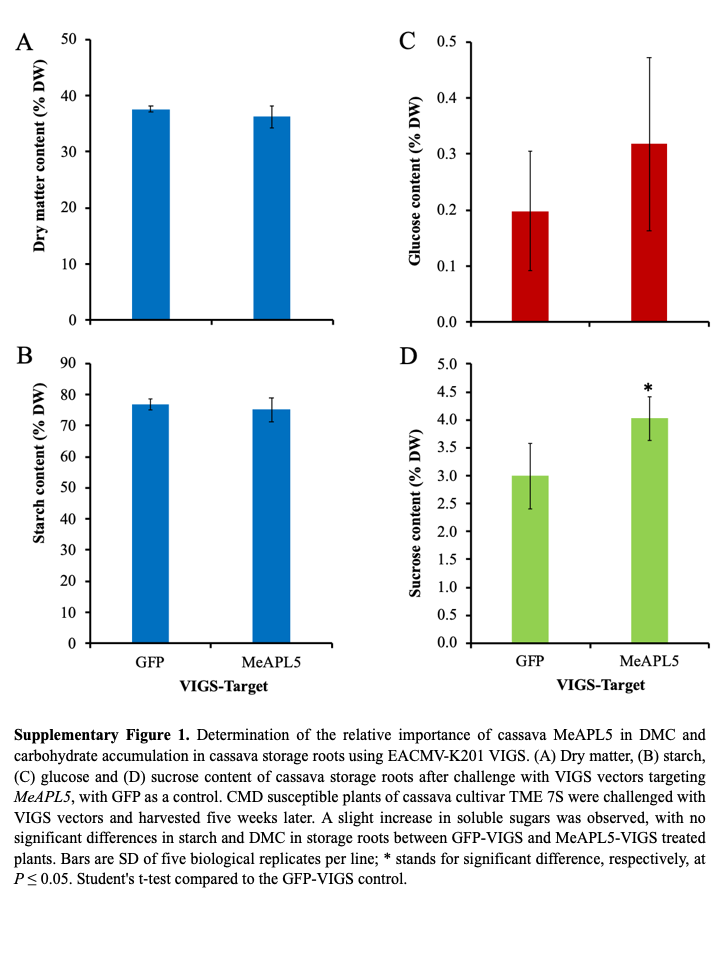

Supplement: Supplementary file 1 — Supplementary Figure 1. Determination of the relative importance of cassava MeAPL5 in DMC and carbohydrate accumulation in cassava storage roots using EACMV-K201 VIGS. (A) Dry matter, (B) starch, (C) glucose and (D) sucrose content of cassava storage roots after challenge with VIGS vectors targeting MeAPL5, with GFP as a control. CMD susceptible plants of cassava cultivar TME 7S were challenged with VIGS vectors and harvested five weeks later. A slight increase in soluble sugars was observed, with no significant differences in starch and DMC in storage roots between GFP-VIGS and MeAPL5-VIGS treated plants. Bars are SD of five biological replicates per line; * stands for significant difference, respectively, at P ≤ 0.05. Student's t‐test compared to the GFP-VIGS control. (TIFF 2028 kb) [file 11103_2020_995_MOESM1_ESM.tiff]

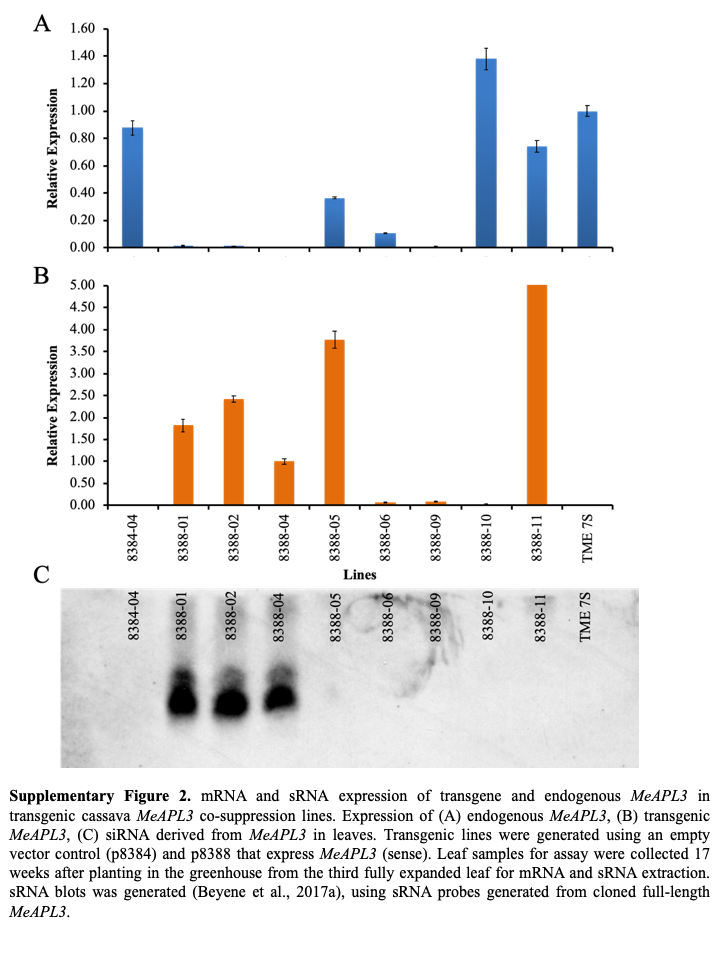

Supplement: Supplementary file 2 — Supplementary Figure 2. mRNA and sRNA expression of transgene and endogenous MeAPL3 in transgenic cassava MeAPL3 co-suppression lines. Expression of (A) endogenous MeAPL3, (B) transgenic MeAPL3, (C) siRNA derived from MeAPL3 in leaves. Transgenic lines were generated using an empty vector control (p8384) and p8388 that express MeAPL3 (sense). Leaf samples for assay were collected 17 weeks after planting in the greenhouse from the third fully expanded leaf for mRNA and sRNA extraction. sRNA blots was generated (Beyene et al., 2017a), using sRNA probes generated from cloned full-length MeAPL3. (TIFF 2028 kb) [file 11103_2020_995_MOESM2_ESM.tiff]
