# Supplementary material for: Cassava shrunken-2 homolog MeAPL3 determines storage root starch and dry matter content and modulates storage root postharvest physiological deterioration
Source: Plant Mol Biol. 2020 Oct 6;109(3):283–99. doi: 10.1007/s11103-020-00995-z (PMC9163024; doi:10.1007/s11103-020-00995-z)
Supplement: Supplementary file 3 — Supplementary Table 1. Primers used for cloning, PCR and RT-qPCR assay of different genes. (PDF 69 kb) [file 11103_2020_995_MOESM3_ESM.pdf]

Supplementary Table 1. Primers used for cloning, PCR and RT-qPCR assay of different genes

Primer #	Primer sequence	Purpose
1302	GAATTCATGGATTCTTGCTGTGTGGC	Cloning of MeAPL3 coding seqyence (EcoRI and BamHI restriction site introduced for subcloning)
1303	TCTGGATCCATTTATATTACTGTGCCA	
1262	GCTAGCGCTCGCAATTCAAGTTTGGA	Cloning of MeAPL1-VIGS targhet (NheI and SbfI restrction sites introduced for subcloning)
1263	CCTGCAGGGTAGTCAAGAAGCGTCTGGT	
1264	GCTAGCGTGGATGCAATTGACTTTGG	Cloning of MeAPL2-VIGS targhet (NheI and SbfI restrction sites introduced for subcloning)
1265	CCTGCAGGGTACCAGCTCCTCCTCCCAGTATAATTG	
1044	GCTAGCCCTAGCAGCAACTCAAACGC	Cloning of MeAPL3-VIGS target (NheI and SbfI restrction sites introduced for subcloning)
1045	CCTGCAGGTAGGGTATCTCCACCGTAGAAGCTT	
1266	GCTAGCCTTTTCCCTCTCACCAAGCG	Cloning of MeAPL4-VIGS targhet (NheI and SbfI restrction sites introduced for subcloning)
1267	CCTGCAGGGAGGCTCGACTGTCATCCATTGGCAAAC	
1489	GATCGTTTTGACATCTTGGTACCATTG	Cloning of MeAPL5 (both cDNA and genomic DNA)
1490	CCTCAGCTTCATCAATGGCATAATC	
1540	GCTAGCTCGTTTGGGTTTTTGAGGATGC	Cloning of MeAPL5-VIGS targhet (NheI and SbfI restrction sites introduced for subcloning)
1541	CCTGCAGGCATTGAATAAATATGCCTGGACATTGTG	
1309	GGGAAGATCAATTTCCCTAGGAAAA	RT-qPCR for MeAPL1-VIGS target
1310	ATAGAGGCAACAGTCTTCGGGTCT	
1311	CTTTACAACTAGTTTAGCTGGCGAGAA	RT-qPCR for MeAPL2-VIGS target
1312	TAAGCCTGTATGCACCTCCAATTG	
1300	TTAATCGGCACCTTGCACGC	RT-qPCR for MeAPL3-VIGS target
1301	CAGCATCTGCAGTTCCTTGGAA	
1313	CTCTTGCTACTAATATAGCTGGCGAATC	RT-qPCR for MeAPL4-VIGS target
1314	ATGCACCTCCAATTGGCACA	
1542	AGGACCACAATGTCCAGGCATATTT	RT-qPCR for MeAPL5-VIGS target
1543	AAACTTTGGTGACTGTTCTGTGAGA	
1475	CACATGAGCGAAACCCTATAGGAAC	RT-qPCR for MeAPL3 transgene (p8388)
1476	CACAATTATAGCAGAGAAGGCGACAA	
1326	CACAAACAAAGATGGTGTGCAAGA	RT-qPCR for MeAPL3 (endogenous gene)
1327	CTTGTACAGCCAACCAGTCCTTGA	
1426	TCTCGACAGACGTCGCGGTGAGTT	PCR primer for transgenic line screen (p8388)
1427	GGATAAGTGCCGGATTGTAGATGCG	
